# Stably Integrating an Inducible CRISPR-Cas9 to Protect Against Viral Infections in Vitro

**DOI:** 10.17912/micropub.biology.000590

**Published:** 2022-06-16

**Authors:** Indeever Madireddy, Merrick Pierson Smela

**Affiliations:** 1 BioCurious, Santa Clara, CA; 2 BASIS Independent Silicon Valley, San Jose, CA; 3 Department of Genetics, Harvard Medical School, Boston, MA

## Abstract

CRISPR-Cas systems protect bacteria from viral nucleic acids. The Cas9 enzyme cleaves bacteriophage DNA preventing viral genes from being expressed in the bacterial host. In this work, the Cas9 protein is repurposed to function as an intracellular mammalian defense mechanism that protects human cells from cytomegaloviral DNA. The A549 lung adenocarcinoma cell line was genetically modified to express a doxycycline-inducible Cas9, and a guide RNA targeting a luciferase reporter plasmid. This investigation revealed a robust inducible Cas9 system that successfully reduced the expression of the luciferase viral reporter by up to 98% and by 75% on average.

**Figure 1. Stable integration of a doxycycline-inducible CRISPR-Cas9 in human cells reduces the expression of viral DNA:**
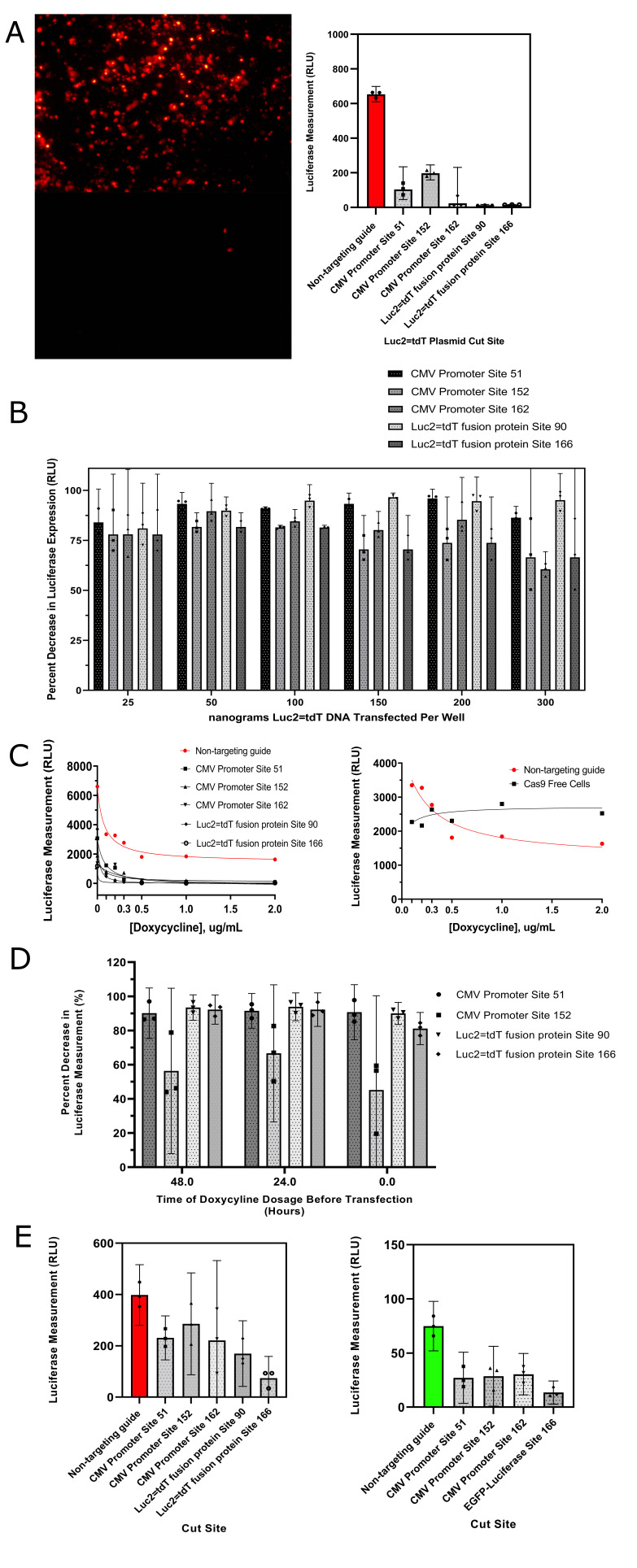
**A)** The top left image shows the transposed A549 cells expressing the non-targeting guide. The bottom left image shows the cells expressing the Luc2=tdT fusion protein site 90 targeting guide. The chart on the right shows luciferase expression measured in relative light units. (p <0.0001 for all cut sites). The red bar depicts a control expressing a guide and Cas9 that do not target the reporter plasmid. 95 percent confidence intervals are also plotted for each bar. All measurements were taken in triplicates and plotted. **B)** The percent decrease in luciferase expression normalized to a non-targeting control for five different guides is shown. The x-axis corresponds to the nanograms of Luc2=tdT reporter DNA transfected into each 96 well, and the y-axis is by what percent expression decreased with Cas9 and a targeting guide. 95 percent confidence intervals are provided. All measurements were taken in triplicates and are plotted on the bar graph. **C)** Luciferase expression versus doxycycline concentration in culture medium during transfection with the Luc2=tdT reporter is shown in the left diagram. The right diagram compares cells expressing the non-targeting guide with Cas9-free cells. **D)** Dosage of 2μg/mL of doxycycline at various time points before transfection of the reporter. **E)** Cas9 was active and expressed during transfection of the two luminescence plasmids. In the left chart, luc2=tdT was cotransfected with Cas9. (p < 0.05 for all cut sites except for CMV Site 152). In the right chart, transfection was also tried with an eGFP-Luciferase C1 fusion protein which was similarly driven by the immediate-early CMV promoter in the same plasmid backbone (p<0.001 for all cut sites).

## Description

Vaccines are currently the most popular and effective method of preventing or reducing the severity of a viral infection (Wagner and Weinberger, 2020). With the advent of vaccinations, diseases such as polio, smallpox, and diphtheria have all been practically eradicated or eliminated (Orenstein and Ahmed, 2017). However, vaccines have limited efficacy in immunocompromised patients. Righi et al. noted that even with vaccination, immunocompromised patients have impaired antibody function or produce low levels of antibodies over time. Eibl and Wolf also noted a reduced response to vaccinations in immunocompromised patients. Hughes et al. found that the influenza vaccine was six times less effective for those who were immunocompromised.

CRISPR-Cas systems could potentially be utilized to reduce the severity of viral infections in immunocompromised patients. CRISPR-Cas systems are found in a variety of bacteria and archaea. These systems originated as a form of bacterial immunity to protect bacteria from bacteriophages (Redman et al., 2016). Cas9 requires a guide RNA (composed of a crRNA and tracrRNA) complementary to the cut site, and the guide target must be directly upstream of an NGG protospacer adjacent motif (PAM).

This work aims to show how the bacterial Cas9 enzyme can be repurposed to act as an inducible intracellular defense mechanism that destroys foreign viral DNA in human adenocarcinoma cells. It is hypothesized that if Cas9 and a guide RNA (gRNA) targeting a viral sequence are stably expressed in these human cells, there will be a reduction in the expression of viral DNA.

Human cytomegaloviruses (HCMV) were chosen as the model viral target as HCMVs are highly prevalent throughout the population, and a majority of people get infected before adulthood (Schottstedt et al., 2010). The virus remains latent in cells, allowing it to spread unnoticed during organ transplants or blood transfusions, which disproportionately affects immunocompromised patients (Griffiths et al., 2020.) Furthermore, no FDA-approved vaccine protects against HCMV infections (Anderholm et al. 2016). Finally, HCMV strains and other viruses in the Herpesviridae family have DNA as their genetic material, making them ideal for testing the efficiency of the DNA targeting Cas9 in eliminating the expression of viral DNA.

The A549 human adenocarcinoma cell line was genetically modified to inducibly express Cas9 and a gRNA that targeted the cytomegalovirus (CMV) immediate-early promoter or a firefly luciferase fusion protein (Luc2=tdT) driven by the same promoter. These two components were expressed by the transient Luc2=tdT plasmid (Patel et al., 2010) (Addgene plasmid # 32904) which was delivered to the A549 cells with Lipofectamine 3000. This plasmid was used as a viral reporter because luciferase expression is easy to quantify. The PiggyBac transposon system was used to integrate a doxycycline-inducible Cas9 and the viral targeting guide RNA (gRNA). qPCR validated integration of both transposons with an average of 2-10 gRNA transposon and 4-14 Cas9 transposon integrations per cell. The RPP30 gene was used as a relative control.

The results supported the robust capabilities of the inducible Cas9. Luminescence and fluorescence of the Luc2=tdT plasmid decreased in the presence of Cas9 (Figure A). Five different sites on the Luc2=tdT plasmid, three within the CMV promoter and two within the fusion protein, were targeted. Luciferase expression decreased by 60-98% depending on the cut site. This indicated that Cas9 was able to effectively protect the cell from the expression of the viral reporter. Targeting the luciferase fusion protein itself tended to be more effective in reducing expression than cleaving the CMV promoter. This suggests that it is still possible to transcribe and express the luciferase reporter protein with a partial promoter though at a reduced level. However, because the Luc2=tdt guides were double cutters, it is more likely the reporter is cleaved and expression decreased in this manner. Furthermore, the results seem to suggest that the Benchling on-target scoring correlates to the guide’s knockout efficiency. Luciferase expression for the CMV promoter guides (CMV162 < CMV 51 < CMV 152) decreased as the on-target score of the guide increased (CMV162 > CMV 51 > CMV 152). This interesting trend lends credibility to the on-target score calculations (Doench et al., 2016).

The viral load tolerance of Cas9 was also assessed (figure B). The A549 cells were transfected with various amounts of plasmid, and overall, Cas9 was consistently able to eliminate expression at a range of viral loads. The 95% confidence intervals tended to overlap between DNA concentrations per guide, and on average, a 75 % decrease in fluorescence across guides and concentrations was observed.

There was also a correlation between doxycycline concentration in the culture medium and luciferase expression. As doxycycline concentration was increased, luminescence tended to decrease (figure C). This suggests that higher doxycycline concentrations lead to more robust activation of Cas9, which in turn significantly decreases luciferase expression. However, the luminescence did not further significantly decrease after doxycycline concentration exceeded 1 µg/mL. The greatest change in luminescence occurred between a concentration of 0 and 0.1µg/mL of doxycycline, which indicated the high sensitivity of the inducible Cas9 system.

One anomalous trend to note is that as doxycycline concentration increased, luminescence tended to decrease for the nontargeting control. This is an interesting trend because doxycycline concentration is expected to not affect luminescence since the nontargeting guide should not be able to cleave viral DNA regardless of how much Cas9 is expressed. Thus there seemed to be a confounding effect of the doxycycline in the culture medium on luciferase expression. However, when tested on cells not containing any transposon, it was found that luminescence remained relatively constant regardless of the doxycycline concentration (figure C). This suggests that a high doxycycline concentration is not the culprit for this decrease and instead, Cas9 production is inhibiting the expression of the luminescence plasmid in the nontargeting control. Either Cas9 is binding to the plasmid at a low level even without a targeting guide or transcribing high levels of Cas9 inherently inhibits the transcription of the reporter plasmid.

The results in figure D suggest that due to the overlap of the 95 percent confidence intervals, there is no relationship between when doxycycline is dosed and how efficiently Cas9 knocked out expression. The results indicate that so long as doxycycline was present, Cas9 was expressed at a high level, decreasing luciferase expression by around 75%. This consistency is similar to the result in figure C and indicates that the inducible Cas9 system is robust and fast-acting.

Luminescence also decreased in the presence of a transiently expressed Cas9 with the same guides. Luciferase expression decreased by 25-80% for the Luc2=tdT plasmid and 60-80% with the EGFP-Luciferase C1 plasmid (Addgene plasmid # 163523). These results (figure E) indicate that transiently transfecting Cas9 will also reduce the expression of the viral DNA and protect the cell. However, for some sites, there was no statistically significant decrease in luciferase expression.

## Methods

*Culturing of A549 Cells*

The A549 (CCL-185) human lung adenocarcinoma cell line was purchased from ATCC (ATCC, Manassas, Virginia). The cell line was cultured in Ham's F-12k nutrient mixture, Kaign’s modification (Corning, Corning, New York) supplemented with 10 percent Fetalgro, L-glutamine, and penicillin/streptomycin. Cells were maintained at 37°C in a humidified incubator at a 5 percent CO _2_ concentration.

*Escherichia Coli Culturing*

*Escherichia Coli (E. coli)* bacteria were regularly used for plasmid cloning and plasmid propagation. *E. coli* were cultured in 2.5 percent LB broth (1 percent tryptone, 0.5 percent yeast extract, 1 percent sodium chloride) or plated on 2.5 percent LB agar which is broth supplemented with 15g/L of agar. *E. coli* containing transposon sequences were grown at 30 °C, a lower temperature than typically used, to prevent unfavorable recombination events. *E. coli* used to propagate or clone non-transposon sequences were grown at the typical 37 °C. 100 µg/mL ampicillin or 50 µg/mL kanamycin were used as a selection marker for the *E. coli* depending on the plasmid.

*gRNA Transposon Cloning for Stable Expression of Cas9*

The gRNAs chosen for targeting were cloned into the PB_rtTA_BsmBI PiggyBac transposon ( Addgene plasmid #126028) (gRNA transposon). This transposon contains a neomycin (G418) resistance cassette for mammalian selection and a reverse tetracycline transactivator that is used to induce Cas9 expression on a separate transposon. A U6 promoter drives expression of the gRNA of interest, and two BsmBI sites flank the cloning region.

*gRNA Design*

gRNAs targeting the CMV promoter and the Luc2=tdT luciferase fusion protein were designed with the CRISPR guide design tool built into the Benchling software. https://benchling.com. Targets were chosen on the basis of having high on target and high off-target scores as determined by Benchling. Three cut sites within the CMV promoter and one target within the Luc2=tdT gene sequence were chosen by these metrics. An additional target in the Luc2=tdT gene was chosen based on previous reports. (Sekine et. al, 2019). Both Luc2=tdT gRNA targets occur twice within the protein sequence, allowing for Cas9 to cut twice with only one guide. One guide was designed for EGFP as well. Table 1 shows the designed targeting guides.

*Transposon Transfection*

A549 cells were grown in 6 well plates to a density of 4.5x10 ^4^ cells/cm ^2^ . Lipofectamine 3000 (Thermofisher, Waltham, Massachusetts) was used to deliver the transposons to the cells. The standard dose lipofectamine protocol was utilized since the high dose was found to have high levels of toxicity to the A549 cells leading to 50 percent cell death. Three plasmids were cotransfected into the cells: the Piggybac transposase, a Cas9 transposon containing an inducible Cas9 (Addgene plasmid # 126029), and the gRNA transposon that expressed the respective gRNA (Schertzer et al., 2019).

*Non-transposon Cloning*

The transient Cas9 plasmid (Addgene plasmid #62988) was cloned with a one-step BbsI Golden Gate method. Plasmids cloned in this work have been deposited to Addgene: https://www.addgene.org/Indeever_Madireddy/

*Luciferase Assay*

The expression of the Luc2=tdt fusion protein allows for the visualization of the cells under a fluorescence microscope and also quantification of the luciferase luminescence in a plate reader. The CMV promoter drives expression of this fusion protein. 2.2x10 ^3^ A549 cells were plated in each well. 50µL of a 500nM Hepes buffer containing 1mg/mL of D-Luciferin was added to each triplicate 96 well in which A549 were plated and transfected. Each well also contained 100µL of culture media. The Tecan 200 PRO Microplate reader was used to collect luciferase measurements from the entire well. Per well readings were taken every 109 seconds over the course of 45 minutes. The last five measurements were averaged to obtain that well’s luciferase measurement in relative light units (RLU) The plate reader was set to luminescence mode with an integration time of 1000ms. Baseline readings were subtracted to remove background fluorescence.

## Reagents

Plasmids obtained for work.

**Table:** 

Plasmid Name	Addgene ID
pSpCas9(BB)-2A-Puro	#62988
eGFP-luciferase-C1	#163523
PB_tre_Cas9	#126029
PB_rtTA_BsmBI	#126028
pcDNA3.1(+)/Luc2=tdT	#32904

qPCR primers.

**Table:** 

Primer Name	Sequence
*RPP30* Forward	AGA TTT GGA CCT GCG AGC G
*RPP30* Reverse	GAG CGG CTG TCT CCA CAA GT
Cas9 Transposon Forward	CAT CGA GCA GAT CAG CGA GT
Cas9 Transposon Reverse	ATC CCG GTG CTT GTT GTA GG
gRNA Transposon Forward	CGG GGC TAA AGT GCA TCT CG
gRNA Transposon Reverse	GAG CGT ACA GTG CGT TCT CC

Reagents and antibiotics used in work.

**Table:** 

Reagent Name	Provider
BbsI-HF	NEB
BsmBI-v2	NEB
T4 DNA Ligase	NEB
Q5 High Fidelity DNA Polymerase Master Mix	NEB
Luna Universal qPCR Mix	NEB
Lipofectamine 3000	Thermofisher
Opti-mem medium	Thermofisher
D-Luciferin	GoldBio
Hygromycin	GoldBio
G418	GoldBio
Doxycycline-Hyclate	GoldBio
Ampicillin Sodium Salt	GoldBio

Organisms used in work.

**Table:** 

Cell	Provider
A549	ATCC
5-alpha *E. coli*	Amid Biosciences

Kits used in this work.

**Table:** 

Kit name	Use
Monarch Genomic DNA Purification Kit	Extracting genomic DNA from A549 cells for qPCR
Monarch Plasmid Miniprep Kit	Purifying plasmids from *E.coli*
